# Report of a Case of Acute Bilateral Psoitis1Read before the Section on Medicine of the Buffalo Academy of Medicine, March 14, 1893.

**Published:** 1893-04

**Authors:** Eugene A. Smith

**Affiliations:** Professor of Anatomy, Medical Department of Niagara University, Buffalo, N. Y.


### REPORT OF A CASE OF ACUTE BILATERAL PSOITIS.[1]

By EUGENE A. SMITH, M. D.,

Professor of Anatomy, Medical Department of Niagara University, Buffalo, N. Y.

The following report of a case of acute inflammation of the psoas magnus muscles is of interest, because of the rarity of acute psoitis, and because the myositis occurred on both sides, and was not due to septic or traumatic causes.

---

1. Read before the Section on Medicine of the Buffalo Academy of Medicine, March 14, 1893.

Several muscular regions may be affected by myositis, the tongue, the psoas, the pectoral and abdominal muscles, and those of the thigh and calf; but the affection is usually a chronic form of inflammation extending by continuity and contiguity of connective tissue structure and secondary to tubercular, syphilitic, rheumatic, septicemia, diathetic conditions, or the presence of parasites. The type of such secondary forms of myositis is psoas abscess, resulting from infiltration and burrowing of pus in the sheath of the psoas magnus, following carious disease of the vertebræ.

Acute glossitis may be instanced as the type of acute myositis, and is more frequent than acute psoitis, because the tongue is more exposed to trauma and especially to injury with septic inoculation.

Suppuration in acute myositis is rare, but is more liable to occur in psoitis.[1] This statement should be modified, I think, to except myositis due to septic causes, which is usually a purulent inflammation.

In the case here reported, the psoitis resulted in resolution, but in the Buffalo Medical and Surgical Journal for December, 1881, Dr. Herman Mynter reported two cases of acute psoitis which terminated in suppuration, requiring operation and the evacuation of large quantities of pus.

On January 12, 1893, C. S., a well-built young man, 21 years old, came to the office; he gave a good family and personal history; he said he worked as a pipe-fitter, but felt obliged to stop working; for ten days he had been having severe pain in the right hypochondriac region, deep-seated, and made worse by a cough; he had lost slightly in weight; appetite not much impaired; tongue slightly furred; bowels regular. On examination, I found him tender to deep pressure in the epigastric and right and left lumbar regions. The pulse was tense and 104, temperature 101° F. On this occasion I prescribed rest, application of hot fomentations, and a mixture of simple elixir with pepsin, the diagnosis being in doubt. In the succeeding five days his temperature did not rise above 102°, and the pulse fell to eighty, becoming softer also under the influence of phenacetine. He perspired freely, daily, usually in the evening. The tenderness became more marked in the lumbar regions, and especially so upon grasping the region of the loin between the hands, one upon the lumbar region in front and the other behind the kidney. I found, also, that the lower limbs were flexed upon the body and rotated outward. The mother noticed that when on his feet he walked with the body bent well for-

1. See "An American Text-Book of Surgery."

ward at the hips. He complained also of pain running down the limbs, especially the left, locating painful points just below Poupart's ligament, and on the inside and outside of the thigh, as far as the knee. The urine was clear, high-colored, strongly acid, Sp. Gr. 1021, and showed no trace of albumen or sugar. Syphilis and lead poisoning excluded, the case presented some features of rheumatic, suppurative or tubercular trouble affecting the psoas muscles. On January 25, 1893, Dr. DeLancey Rochester saw the patient and the following additional history was obtained. Just after Christmas he had run some distance and then stood watching a large fire, and upon returning home felt chilled; shortly after, he began having the pain he first complained about. Upon a second visit Dr. Rochester advised an alkaline diuretic, which corrected the acidity of the urine, and in the next five days the fever ranged between 99° F. and 100° F. On January 31st, the temperature again ran up to 102½° F., and I found a soft mitral regurgitant murmur to account for it, while the lumbar tenderness was less marked and muscular contraction also. Changing the prescription to salicylate of sodium with bicarbonate of sodium, and keeping him at rest, were the features of the treatment until after a normal temperature for ten days. I considered it safe to allow him to walk. During the time when tubercular trouble with pus formation was a question of diagnosis, deep pressure over the psoas magnus muscles along the front of the spine was quite painful, also just behind the kidneys, between them and the spine.

In this case the diagnosis of acute psoitis, rheumatic in origin and affecting both muscles, was made after the endocarditis appeared. Before that, it was a question of differential diagnosis strongly pointing to suppurative psoitis of probable tubercular origin and calling for a radically different treatment.

